# Deciphering Genetic Architecture of Feed Conversion Ratio and Growth Traits in Yorkshire Pig

**DOI:** 10.3390/genes17030289

**Published:** 2026-02-27

**Authors:** Changguang Lin, Qiuyong Chen, Yaxuan Liu, Wei Cai, Tao Huang, Yi Zhou, Jinyu Lin, Lunjiang Zhou, Xinzhu Chen

**Affiliations:** 1 Institute of Animal Husbandry and Veterinary Medicine, Fujian Academy of Agricultural Sciences, Fuzhou 350013, China; fjchenqiuyong@163.com (Q.C.); lunjiang@163.com (L.Z.); 2 Fujian Guanghua Best Ecological Agriculture and Animal Husbandry Development Company Limited, Youxi 365106, China; liuyaxuan8@163.com (Y.L.); zytobe1@163.com (Y.Z.); 13950411983@163.com (J.L.); 3 Youxi County Bureau of Agriculture and Rural Affairs, Youxi 365106, China; 545caiwei@163.com (W.C.); yxsylsc@126.com (T.H.)

**Keywords:** feed efficiency, genome-wide association study, genetic parameters, pig, genomics

## Abstract

Background: Pigs are one of the most important livestock species for providing meat products in the world. Deciphering the genetic architecture of feed efficiency-related traits is beneficial to improve the genetic progress of these traits and save the total cost of pork production. However, the genetic architecture of feed efficiency-related traits remains unclear. Methods: To address this problem, we collected 1301 genotyped Yorkshire pigs with three feed efficiency-related traits, including days at 100 kg (DAYS_100), backfat thickness at 100 kg (BFT_100), and feed conversion ratio from 30 to 100 kg (FCR_30_100), to explore the genetic parameters and genetic basis of these traits. Results: The heritability of DAYS_100, BFT_100, and FCR_30_100 was 0.25 ± 0.04, 0.40 ± 0.05, and 0.23 ± 0.04, respectively. Additionally, BFT_100 and DAYS_100 had a weak negative genetic correlation (−0.01 ± 0.12), while trait FCR_30_100 showed a positive genetic correlation with DAYS_100 (0.51 ± 0.11) and BFT_100 (0.28 ± 0.12). A genome-wide association study identified 7, 5, and 4 SNPs independently associated with BFT_100, DAYS_100, and FCR_30_100, respectively. Further analysis found that the candidate gene *ETV4* was significantly associated with DAYS_100 and the candidate gene *ENSSSCG00000045751* was associated with FCR_30_100. The functional annotation of candidate genes was enriched in the bile acid metabolic process and protein ubiquitination terms. Conclusions: This study discovered 16 quantitative trait loci associated with feed efficiency-related traits, providing a comprehensive insight for understanding the genetic basis of feed efficiency-related traits in pigs. The candidate genes, such as *ETV4* gene in DAYS_100, *CAMK1D* gene for BFT_100, and *ENSSSCG00000045751* gene for FCR_30_100, could be used for further investigation.

## 1. Introduction

Pigs are one of the most important livestock species for providing meat products in the world. In the process of pig production, feed occupies about 65% of the total cost of pork production [1]. Consequently, improving feed efficiency is a primary objective in swine breeding programs. Key growth traits, such as days at targeted body weight (DAYS) and backfat thickness (BFT), are routinely used to evaluate production performance in pigs because these traits are easy to measure [2]. Additionally, the feed conversion ratio (FCR), defined as the amount of feed consumed per unit of body weight gain, serves as a critical indicator of feed efficiency. A lower FCR not only reduces production costs but also decreases nitrogen and phosphorus excretion, thereby contributing to environmental sustainability [3]. However, large-scale phenotypic recording of individual feed intake has been challenging, limiting genetic evaluation of FCR. Recent advances in automated feeding systems now enable precise, high-throughput measurement of individual feed consumption (e.g., Osborne Feed Intake Recording Equipment system and Nedap Velos system), offering opportunities to dissect the genetic architecture underlying feed efficiency in pigs.

Genetic parameters of complex traits are fundamental to understanding the genetic nature of complex traits. Previous studies have shown that feed efficiency traits are genetically correlated with growth traits [4], conformation traits [5], reproduction traits [6], and others [7,8,9], indicating the genetic links between FCR and other complex traits. A genome-wide association study (GWAS) provides chances to reveal the genetic architecture of complex traits and diseases in humans and farm animals [10,11]. For example, previous studies found that several genomic regions were associated with feed efficiency traits in pigs [4,12,13]. However, these studies only used the low-coverage data to explore the genetic basis of feed efficiency traits in pigs. The strategy of genotype imputation provided chances to improve the genome coverage of genotype data and identify putative causal variants associated with complex traits [14,15]. For example, the SWine IMputation haplotype reference panel improved the genotype resolution for genetic mapping in pigs [16]. These studies provided a comprehensive view on exploring the genetic architecture of feed efficiency-related traits in pigs.

Therefore, to elucidate the genetic basis of feed efficiency-related traits in pigs, we collected 1301 genotyped Yorkshire pigs with three feed efficiency-related traits, including days at 100 kg (DAYS_100), backfat thickness at 100 kg (BFT_100), and feed conversion ratio from 30 to 100 kg (FCR_30_100). First, we estimated the genetic parameters of these feed efficiency-related traits, including heritability and genetic correlation. Subsequently, we performed GWAS to investigate the genetic basis of feed efficiency-related traits. Finally, to investigate the potential function of candidate genes for feed efficiency-related traits, we annotated the candidate genes of GWAS signals and performed functional annotation for the candidate genes.

## 2. Materials and Methods

### 2.1. Population and Data

We collected a Yorkshire breeding population, including 1301 sows derived from Fujian Guanghua Best Limited Company. All sows were manipulated under uniform nutritional and management conditions. All sows were fed until a weight of around 100 kg for each pig. All sows were housed in groups equipped with an automated performance testing system, Osborne Feed Intake Recording Equipment system (Version 2.2.1.7 for Windows; Osborne Industries Inc., Osborne, KS, USA). The system utilized radio-frequency identification (RFID) via electronic ear tags for individual recognition and precisely recorded individual feed intake throughout the trial period. The feeding data were automatically collected by the system and used for the subsequent calculation of the feed conversion ratio (FCR). The basal diet, primarily composed of corn and soybean meal, was formulated to meet the nutrient requirements recommended by the NRC (2012) [17]. All pigs were fed the same diet during the experimental period.

Samples were collected from the ear tissue of each experimental pig. After collection, samples were placed in centrifuge tubes containing 70% ethanol for storage. For genotype data, a total of 1301 pigs were genotyped using Zhongxin No.1 50K SNP Chip and obtained 45,073 single-nucleotide polymorphisms (SNPs). To ensure the genotype quality, we performed the quality control using plink v1.90 [18]. After quality control, the genotypes were imputed from 50 K to whole-genome sequence (WGS) resolution based on the Pig Genomics Reference Panel using Beagle v5.1 [19] with parameters: ne = 1000. After that, we filtered out the low-quality SNPs with multiple allelic SNPs, dosage R-square < 0.8, or minor allele frequency <0.01. Finally, 10,401,626 SNPs were kept for downstream analysis.

For phenotype data, we collected three phenotypes, including days at 100 kg (DAYS_100), backfat thickness at 100 kg (BFT_100), and feed conversion ratio from 30 to 100 kg (FCR_30_100). DAYS_100 was defined as the number of days from birth until the body weight reached 100 kg. BFT_100 was measured via ultrasound at the location between the third and fourth last ribs when the body weight reached 100 kg. FCR_30_100 was evaluated from 30 kg to 100 kg. The physiologically implausible records for each trait were excluded, representing the records exceeding 3 standard deviations.

### 2.2. Estimation of Genetic Parameters

To investigate the genetic parameters of these growth traits, we estimated narrow-sense heritability ($h^{2}$) using GCTA v1.94.1 [20]. Additionally, we utilized a bivariate linear mixed model to calculate the genetic correlation between these growth traits. The linear mixed model was as follows:

y=Xb+Zg+e

where y is a vector of phenotypic records. b is the fixed effect, including year and season. g~N(0, **G**$\sigma_{g}^{2}$) is the additive genetic effect. **G** is the genomic relationship matrix and $\sigma_{g}^{2}$ is the additive genetic variance. e~N(0, **I**$\sigma_{e}^{2}$) is the residual effect. **I** is the identity matrix and $\sigma_{e}^{2}$ is the residual variance. X and Z are the design matrices for the fixed effect b and genetic additive effect g.

### 2.3. Genome-Wide Association Studies (GWAS)

We performed genome-wide association studies for each growth trait using GCTA v1.94.1 [20]. The linear mixed model was as follows:

$$y=x_{i}\beta_{i}+Xb+Zg+e$$

where y is a vector of phenotypic records. $x_{i}$ is a vector of mean-centered genotypes of the SNP i coding as 0, 1, and 2. $\beta_{i}$ is the effect size of SNP i. b is the fixed effect, including year and season. g~N(0, **G**$\sigma_{g}^{2}$) is the additive genetic effect. G is the genomic relationship matrix and $\sigma_{g}^{2}$ is the additive genetic variance. e~N(0, **I**$\sigma_{e}^{2}$) is the residual effect. I is the identity matrix and $\sigma_{e}^{2}$ is the residual variance. X and Z are the design matrices for the fixed effect b and genetic additive effect g. We considered *p* < 5 × 10^−4^ as the significant threshold to obtain significant SNPs.

### 2.4. Conditional Analysis

To obtain the independent SNPs for these growth traits, we conducted conditional analysis based on the GWAS summary statistics for each growth trait using GCTA v1.94.1 COJO module [21]. We utilized the genotype data as the LD reference panel. For the independent variants, we performed a variant effect predictor to predict the variant functional location searching by the interface in Ensembl (URL: https://asia.ensembl.org/index.html, Sus scrofa 11.1, version 114).

### 2.5. Candidate Genes and Functional Annotation

We defined the quantitative trait loci (QTL) based on the independent SNPs. We defined 1 Mb up- and downstream around the independent SNPs as the QTL. The candidate genes located in the QTL region were annotated based on the pig genome annotation (Sus scrofa 11.1) using BEDtools v2.31.1 [22]. Gene Ontology (GO) enrichment analysis and Kyoto Encyclopedia of Genes and Genomes (KEGG) pathway analysis of candidate genes were conducted using DAVID (URL: https://davidbioinformatics.nih.gov/) [23,24].

## 3. Results

### 3.1. Phenotypic Summary and Genetic Parameters of Three Traits

The summary statistics of phenotype records are shown in Table S1. The distribution of phenotypic data essentially conforms to a normal distribution (Figure S1). In addition, we found that the average of FCR_30_100 was 2.29 ± 0.23, indicating the relatively moderate efficiency in this population. To investigate the genetic parameters of these growth traits, we estimated the narrow-sense heritability for these growth traits (Table 1). The heritability of DAYS_100, BFT_100, and FCR_30_100 was 0.25 ± 0.04, 0.40 ± 0.05, and 0.23 ± 0.04, respectively, indicating moderate-to-high heritability across these growth traits. To further exploit the genetic relationship between these traits, we estimated the genetic and phenotypic correlation across these traits. We found that the genetic correlation of these traits was positive (Table 2). Meanwhile, BFT_100 and DAYS_100 had a weakly negative genetic correlation (−0.01 ± 0.12). Additionally, we found that trait FCR_30_100 showed a positive genetic correlation with DAYS_100 (0.51 ± 0.11) and BFT_100 (0.28 ± 0.12), indicating moderate-to-strong correlation between these traits.

### 3.2. Genome-Wide Association Study (GWAS) for Three Growth Traits

**Figure 1:**
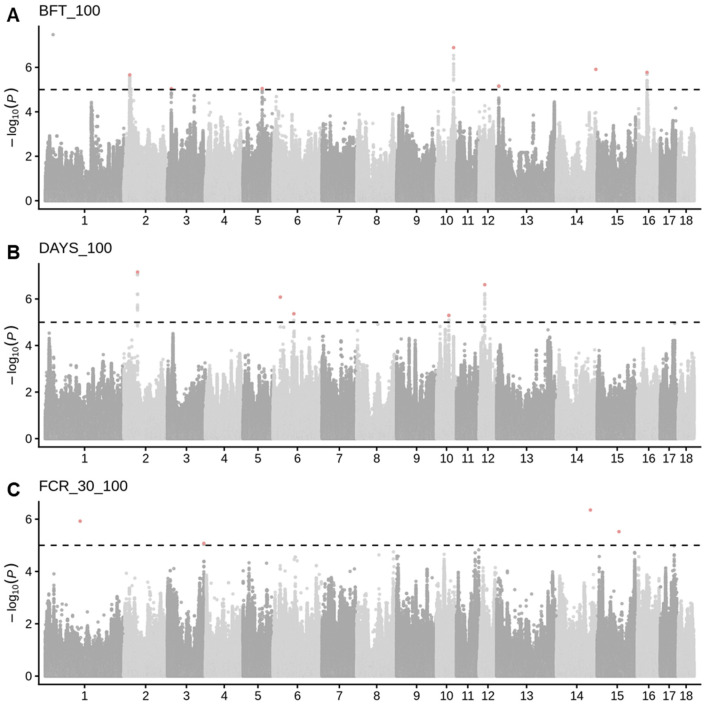
The Manhattan plot of the genome-wide associated study for three growth traits. (**A**) DAYS_100, (**B**) BFT_100, (**C**) FCR_30_100. The black dashed line indicated the suggestive significant threshold (*p* < 5 × 10^−4^). The red points represent the significantly independent SNPs derived from conditional analysis.

To identify SNPs associated with three growth traits, we perform GWAS for each trait. In total, we identified that 114, 90, and 5 were significantly associated with BFT_100 (Figure 1A), DAYS_100 (Figure 1B), and FCR_30_100 (Figure 1C), respectively. After conditional analysis, 7, 5, and 4 SNPs were independently associated with BFT_100, DAYS_100, and FCR_30_100, respectively.

**Figure 2:**
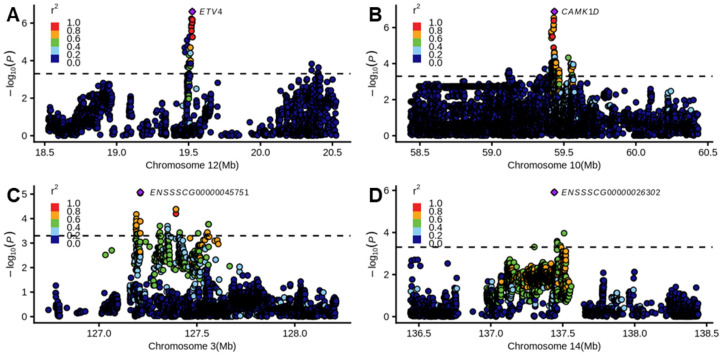
Examples of local Manhattan plots of the genome-wide associated study. (**A**) The local Manhattan plot of the *ETV4* gene associated with DAYS_100 on chromosome 12. (**B**) The local Manhattan plot of the *CAMK1D* gene associated with BFT_100 on chromosome 10. (**C**) The local Manhattan plot of the *ENSSSCG00000045751* gene associated with FCR_30_100 on chromosome 3. (**D**) The local Manhattan plot of the *ENSSSCG00000026302* gene associated with BFT_100 on chromosome 14. The color of points indicates the linkage disequilibrium between the lead SNP and the corresponding SNP. The black dashed line indicated the suggestive significant threshold (*p* < 5 × 10^−4^). The purple rhombus indicates the lead SNP of corresponding GWAS signal.

Furthermore, to identify the candidate genes of independent SNPs, we annotated the nearest genes for each QTL, and several candidate genes were found that are associated with growth traits (Table 3). For example, the nearest gene *ETV4* was significantly associated with DAYS_100 on chromosome 12 (Figure 2A). The nearest gene *CAMK1D* was significantly associated with BFT_100 on chromosome 10 (Figure 2B). Moreover, we discovered several new genes associated with three growth traits. For example, the unannotated candidate gene *ENSSSCG00000045751* was associated with FCR_30_100 on chromosome 3 (Figure 2C). The unannotated candidate gene *ENSSSCG00000026302* was associated with BFT_100 (Figure 2D). In summary, the genome-wide association studies discovered several candidate genes associated with the feed conversion ratio and growth traits.

### 3.3. Functional Annotation Enrichment of Candidate Genes

To identify the candidate genes of three growth traits, we defined quantitative trait loci (QTL) for each independent SNP and annotated the list of candidate genes (Table 3). After that, we performed functional annotation of candidate genes for each trait. We found that the candidate genes of BFT_100 were enriched in biological processes and immunological pathways, such as protein maturation, negative regulation of oxidoreductase activity, and cytotoxic T cell pyroptotic cell death (Table 4), indicating the potential links between immunological processes and the formation of backfat. In addition, the candidate genes of DAYS_100 were enriched in translation and microvillus membrane terms (Table 4), representing the importance of basic biological processes in growth. Notably, we found that the candidate genes of FCR_30_100 were enriched in bile acid metabolic processes and protein ubiquitination terms, indicating the importance of energy metabolism in the process of feed conversion in pigs (Table 4). In summary, the candidate genes associated with feed efficiency-related traits played a vital role in the important biological processes.

## 4. Discussion

In this study, we systematically exploited the genetic parameters of feed efficiency-related traits. The results show that these feed efficiency-related traits had moderate-to high-heritability. D. N. Do et al. [25] found that the heritability of RIF in Yorkshire pigs was 0.39, and there was a high genetic correlation with FCR (from 0.76 to 0.99), indicating the high genetic links between RFI and FCR. A recent study found that the heritability of feed efficiency-related traits ranged from 0.13 to 0.36 [4]. In addition, we investigated the genetic correlation between FCR_30_100, DAYS_100, and BFT_100. Among these, DAYS_100 and BFT_100 showed a low genetic correlation. In this study, we found that FCR was moderately correlated with DAYS_100 (0.51 ± 0.11) and BFT_100 (0.28 ± 0.12). Tusingwiire et al. [26] displayed a low-to-moderate genetic correlation between daily feed intake and economically important traits (ranging from 0.04 to 0.29). Coyne et al. [27] also found that body weight was positively correlated with feed efficiency in pigs. These results indicated the moderate-to-high links between FCR and key growth traits.

We further explored the genetic architecture of feed efficiency-related traits using GWAS. In this study, we discovered a series of candidate genes associated with feed efficiency-related traits. For example, candidate gene *ETV4* was significantly associated with DAYS_100 on chromosome 12, which was associated with adipose deposition [28], supporting the links between this gene and DAYS_100. Additionally, candidate gene *CAMK1D* was significantly associated with BFT_100. Precious studies also found that *CAMK1D*, a genetic hotspot in type 2 diabetes, might be linked to the activation of food intake and metabolic regulation [29,30]. Moreover, GWAS of FCR discovered that the novel candidate gene *ENSSSCG00000045751* was associated with FCR_30_100, which indicated the potential regulation of feed efficiency. A meta-analysis systematically identified the candidate genes associated with feed efficiency traits, highlighting that the genes *MED18*, *PHACTR4*, and *ABCC2* are strong candidates for FCR [31]. In addition, the functional enrichment analysis showed that the candidate genes associated with feed efficiency and feed efficiency-related traits were highly enriched in the biosynthesis, digestion, and metabolism of biomolecules [4]. In this study, the candidate genes of FCR_30_100 were also associated with the bile acid metabolic process and protein ubiquitination terms, indicating the potential function of these candidate genes.

In this study, we investigated the genetic parameters and genetic basis of feed efficiency-related traits, including FCR_30_100, DAYS_100, and BFT_100. Firstly, we investigated the genetic parameters of feed efficiency-related traits, which provide comprehensive insights into the genetic characteristics of these traits. Additionally, compared with previous studies, this study utilized the imputed genotype data to perform GWAS, which partially improved the discovery rate of SNPs associated with feed efficiency-related traits. Furthermore, this study investigated the potential biological function of candidate genes for each trait and found that the candidate genes played a vital role in the energy metabolic processes. These results contributed to our understanding of the genetic basis of feed efficiency-related traits. In fact, except for the genetic impact, the environmental factors also influenced the feed efficiency-related traits. For example, the composition of the gut microbiome was correlated with the feed efficiency, which indicates the contribution of the microbial community for shaping host productive parameters [12,32]. In addition, this study lacks experimental validation of the candidate genes. Additionally, multi-omics data provided comprehensive insights into understanding the potential genetic regulation underlying complex traits [33]. Therefore, future studies might be necessary to consider the impact of the environment, integrate the multi-omics data, and perform the experimental validation of the candidate genes.

## 5. Conclusions

This study revealed the genetic basis of feed efficiency-related traits. The candidate genes, such as the *ETV4* gene in DAYS_100, the *CAMK1D* gene for BFT_100, and the *ENSSSCG00000045751* gene for FCR_30_100, could be used for further investigation.

## Figures and Tables

**Table 1:** The genetic component and heritability of three growth traits.

Trait Name	$\sigma_{g}^{2}$	$\sigma_{e}^{2}$	$\sigma_{p}^{2}$	h^2^	S.E. of h^2^
BFT_100	2.292	3.450	5.742	0.399	0.046
DAYS_100	17.758	53.341	71.099	0.250	0.043
FCR_30_100	0.010	0.032	0.042	0.232	0.042

Note: $\sigma_{g}^{2}$, $\sigma_{e}^{2}$ and $\sigma_{p}^{2}$ indicate additive genetic variance, residual variance, and phenotypic variance, respectively. h^2^ represents narrow-sense heritability. S.E., standard error.

**Table 2:** The genetic and phenotypic correlation between three growth traits.

Trait Name	DAYS_100	BFT_100	FCR_30_100
DAYS_100		−0.17	0.49
BFT_100	−0.01 ± 0.12		0.11
FCR_30_100	0.51 ± 0.11	0.28 ± 0.12	

Note: The upper triangle represents phenotypic correlations between pair-wise traits. The ones at a lower triangle represent genetic correlations following the standard error of genetic correlations between three growth traits.

**Table 3:** The list of the closest candidate genes for each quantitative trait loci.

Trait Name	Lead SNP	QTL_LEFT (bp)	QTL_RIGHT (bp)	Nearest Gene
BFT_100	10_59434057_G_T	59,413,624	59,566,411	*ENSSSCG00000011111*
BFT_100	13_7171260_T_A	7,170,453	7,321,390	*ENSSSCG00000011207*
BFT_100	14_137441397_A_T	137,064,258	137,543,488	*ENSSSCG00000026302*
BFT_100	16_33918041_G_A	33,690,212	34,917,782	*ENSSSCG00000031337*
BFT_100	2_20431644_T_C	19,438,303	20,508,148	*ENSSSCG00000063305*
BFT_100	3_13730890_T_C	13,725,012	14,532,164	*ENSSSCG00000007727*
BFT_100	5_66986479_A_G	66,864,945	67,408,784	*ENSSSCG00000000735*
DAYS_100	10_42834620_C_T	41,839,723	43,613,996	*ENSSSCG00000011028*
DAYS_100	12_19525840_A_T	19,325,840	19,725,840	*ENSSSCG00000017379*
DAYS_100	2_47777593_C_T	47,735,590	48,022,600	*ENSSSCG00000039410*
DAYS_100	6_25791890_A_T	25,697,308	25,912,238	*ENSSSCG00000002795*
DAYS_100	6_73050130_T_C	73,007,186	73,820,062	*ENSSSCG00000003451*
FCR_30_100	1_121609964_C_G	121,548,294	121,632,035	*ENSSSCG00000004643*
FCR_30_100	14_118563486_G_C	118,363,486	118,763,486	*ENSSSCG00000054704*
FCR_30_100	15_76433998_C_G	75,451,476	76,494,642	*ENSSSCG00000032177*
FCR_30_100	3_127211208_T_C	127,186,275	127,609,263	*ENSSSCG00000045751*

**Table 4:** The functional annotation of candidate genes for each trait.

Trait_Name	Type	Term	Counts	*p*
BFT_100	BP	cytotoxic T cell pyroptotic cell death	2	0.00307
	BP	protein maturation	3	0.00322
	BP	negative regulation of oxidoreductase activity	2	0.0046
	BP	multi-ciliated epithelial cell differentiation	2	0.0046
	BP	granzyme-mediated programmed cell death signaling pathway	2	0.0137
	BP	positive regulation of double-strand break repair	2	0.0183
	CC	nucleus	16	0.00547
	MF	RNA helicase activity	3	0.00355
	MF	hydrolase activity, acting on acid anhydrides, in phosphorus-containing anhydrides	2	0.0135
	MF	serine-type endopeptidase activity	3	0.0327
	MF	chromatin extrusion motor activity	2	0.0486
	MF	ATP-dependent H3–H4 histone complex chaperone activity	2	0.0486
DAYS_100	BP	translation	3	0.0489
	CC	microvillus membrane	2	0.0211
FCR_30_100	BP	bile acid metabolic process	2	0.00929
	BP	protein ubiquitination	3	0.0267
	BP	positive regulation of translation	2	0.0432
	BP	RNA processing	2	0.044
	CC	BBSome	2	0.00651

Note: BP, biological process. CC, cellular component. MF, molecular function. DAYS_100, days at 100 kg, BFT_100, backfat thickness at 100 kg, and FCR_30_100, feed conversion ratio from 30 to 100 kg.

## Data Availability

The data that support the findings of this study are available on request from the corresponding author.

## Supplementary Materials

The following supporting information can be downloaded at: https://www.mdpi.com/article/10.3390/genes17030289/s1, Figure S1: The distribution of phenotype data across three growth traits; Table S1: The summary statistics of phenotype data across three growth traits.

## Abbreviations

The following abbreviations are used in this manuscript:

GWAS	Genome-wide association study
QTL	Quantitative trait locus
SNP	Single-nucleotide polymorphism
WGS	Whole-genome sequence
BFT	Backfat thickness
BFT_100	Backfat thickness at 100 kg
DAVID	Database for Annotation, Visualization and Integrated Discovery
DAYS	Days at targeted body weight
DAYS_100	Days at 100 kg
FCR	Feed conversion ratio
FCR_30_100	Feed conversion ratio from 30 to 100 kg
GRM	Genomic relationship matrix
LD	Linkage disequilibrium
MAF	Minor allele frequency
GO	Gene Ontology
KEGG	Kyoto Encyclopedia of Genes and Genomes
BP	Biological process
CC	Cellular component
MF	Molecular function
