# Supplementary material for: Deciphering Genetic Architecture of Feed Conversion Ratio and Growth Traits in Yorkshire Pig
Source: Genes (Basel). 2026 Feb 27;17(3):289. doi: 10.3390/genes17030289 (PMC13026285; doi:10.3390/genes17030289)
Supplement: Supplementary file 1 [file genes-17-00289-s001.zip › genes-4176587-supplementary.pdf]

Supplementary Material

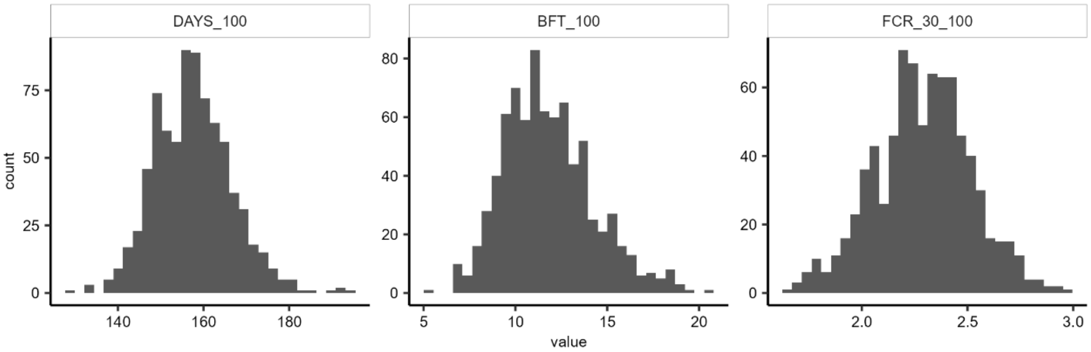

Figure S1. The distribution of phenotype data across three growth traits.

Table S1. The summary statistics of phenotype data across three growth traits.

	**records**	**Min**	**Median**	**Mean**	**SD**	**Max**	**CV**
DAYS_100	1301	129.08	157.47	157.81	8.64	194.6	5.48
BFT_100	1301	5.03	11.61	11.85	2.42	22.01	20.46
FCR_30_100	1301	1.75	2.302	2.3	0.22	2.975	9.55
